# Wilhelm von Waldeyer-Hartz—A Great Forefather: His Contributions to Anatomy with Particular Attention to “His” Fascia

**DOI:** 10.3389/fsurg.2017.00074

**Published:** 2017-12-04

**Authors:** Hubert Scheuerlein, Frank Henschke, Ferdinand Köckerling

**Affiliations:** ^1^Department for General and Visceral Surgery, St. Vincenz Hospital, Paderborn, Germany; ^2^Pathology Paderborn, Paderborn, Germany; ^3^Department of Surgery and Center for Minimally Invasive Surgery, Vivantes Hospital, Berlin, Germany

**Keywords:** Waldeyer fascia, surgical anatomy, pelvis, neuron, chromosome

## Abstract

Wilhelm Waldeyer was, at his time, one of the most well-known authors in the field of Anatomy, Pathology, and Embryology. He held various distinguished academic positions. He was Professor of (Pathological) Anatomy in Breslau, Strasbourg, and Berlin. He remained in Berlin for the unusually long period of 33.5 years, as Full Professor for Anatomy and Director of the Anatomical Institute. His great talent as a teacher ensured that his lectures were always filled to the brim. Between 1862 and 1920, he published 270 works, including classics such as “Das Becken” (The Pelvis). The portrayal of this most important area is counted as one of the most complete which has ever been accomplished in the field of topographic anatomy, it includes the description of the fascia of Waldeyer. He also coined the phrases “chromosome” and “neuron” with their anatomical–morphological concepts. Already during his lifetime, his teaching ability significantly preceded the research capacity. It would, however, be false to overshadow Waldeyer’s merits as a researcher. His main scientific merit is in his excellent summarizing interpretations of current questions of anatomy and evolution, which particularly shows his simultaneous gift as a researcher and a teacher.

## Introduction

**Figure 1:**
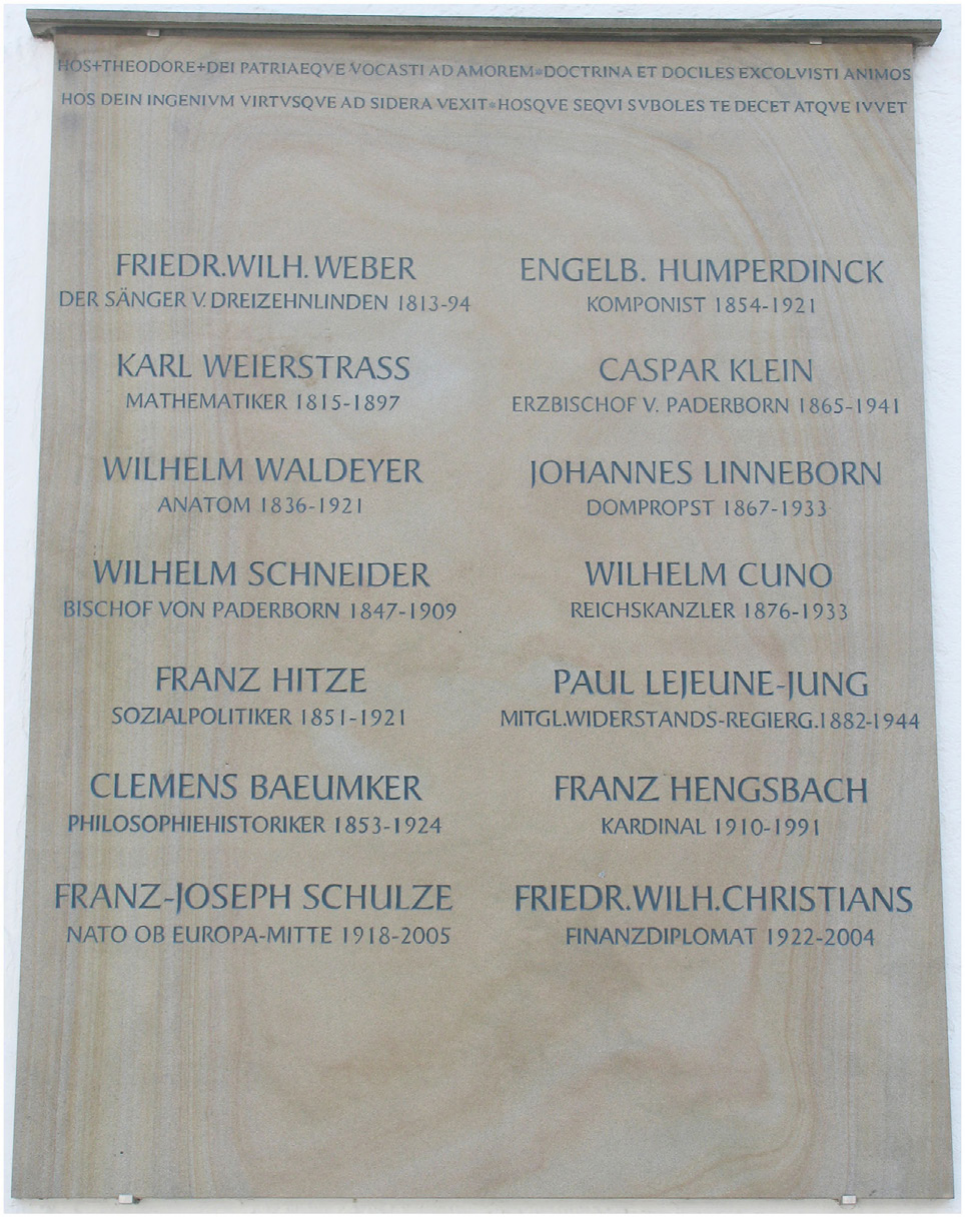
Memorial tablet of famous alumni of the Gymnasium Theodorianum, Paderborn.

Heinrich Wilhelm Gottfried Waldeyer, known as Wilhelm von Waldeyer-Hartz from 1916, was born in Hehlen an der Weser in 1836 and died in Berlin in 1921. He attended the Gymnasium Theodorianum in Paderborn (Figure 1); he is not to be mistaken for his great-nephew Anton Waldeyer (1901–1970) who was born in Tietelsen (Kreis Höxter) and also attended the Theodorianum in Paderborn [after periods of residence in Münster, Berlin, Würzburg, München, Kiel, Freiburg, and Shanghai, Anton Waldeyer ultimately ended up in Berlin and from 1954 he became Full Professor for Anatomy in the Medizinische Fakultät of the Humboldt-Universität (1)].

**Figure 2:**
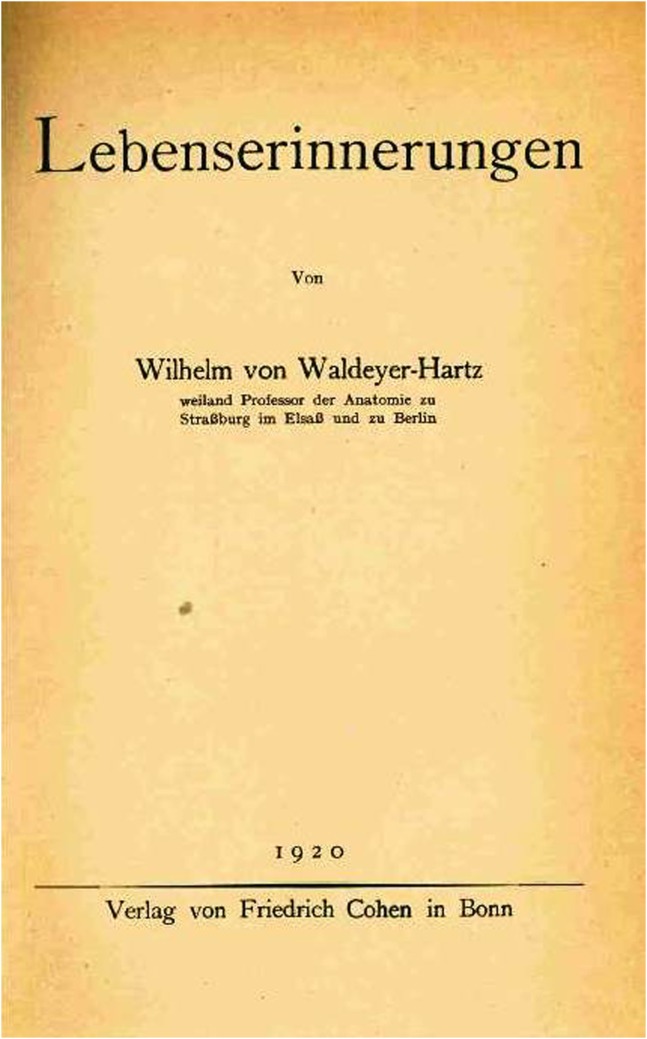
Front page of Waldeyers memoirs (photo).

**Figure 3:**
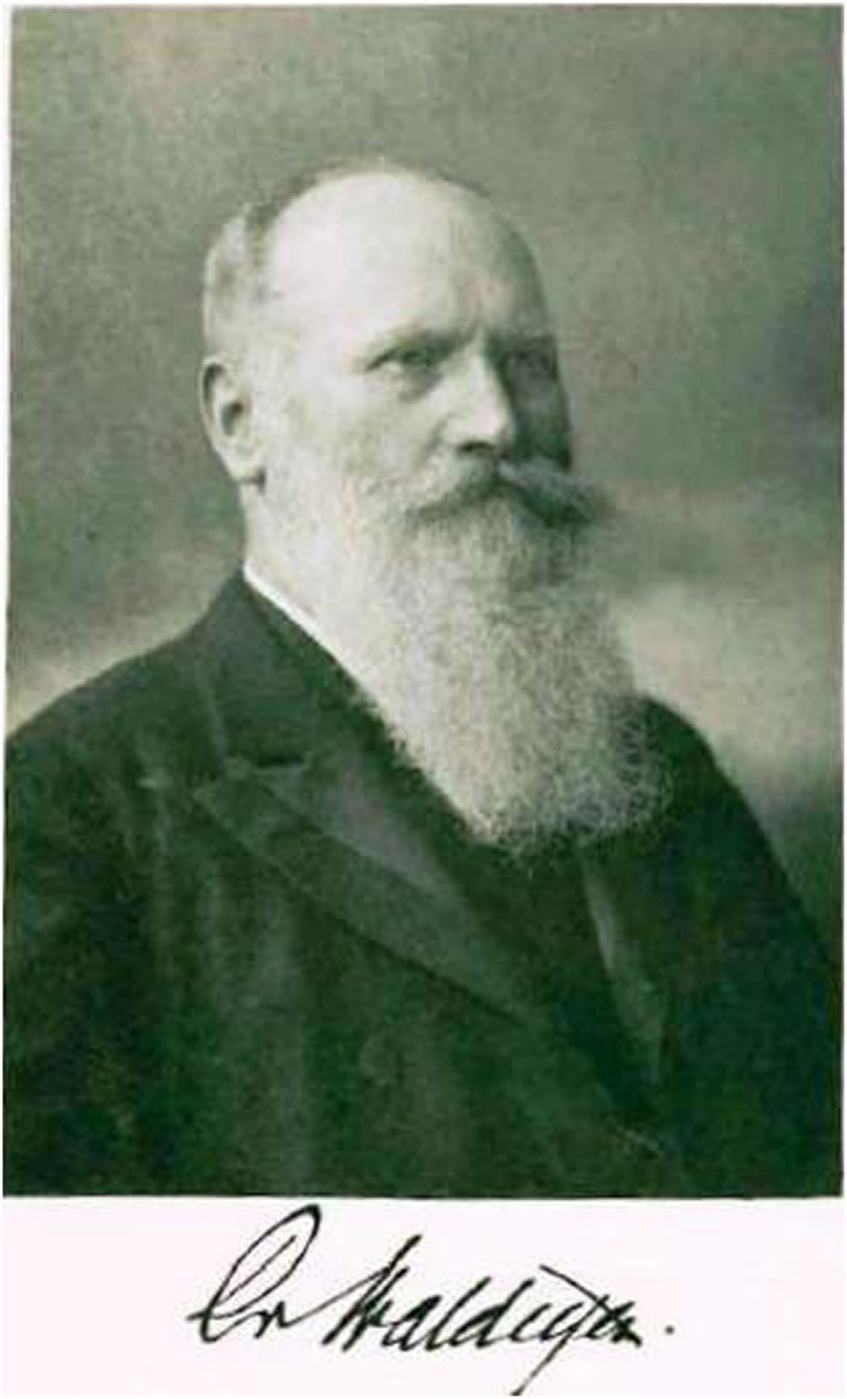
Portrait and signature of Waldeyer (photo, inside the book cover of the Memoirs).

Wilhelm von Waldeyer-Hartz was, at his time, one of the most well-known authors in the field of Anatomy, Pathology, and Embryology and held various distinguished academic positions, especially as Professor of (Pathological) Anatomy in Breslau, Strasbourg, and Berlin (2, 3). Between 1862 and 1920, he published 270 works, including classics such as “Das Becken” [The Pelvis, 1899 (4, 5)]. These works encompass a further thematic medley and deal with subjects such as the problem of cancer, retro-peritoneal hernias, the discovery of the ovarian germinal epithelium, the lymphatic pharyngeal ring, the topographic relationship of the pregnant uterus, the neuron theory, and the pelvic viscera. Many of these are actual pioneer works. The terms “Waldeyer’s fascia” and “Waldeyer’s lymphatic ring” are still found today in practically every textbook of anatomy. He coined the phrases “chromosome” and “neuron” with their anatomical–morphological concepts. Numerous terms which are less common today are associated with his name: he systemized the labels of the appendix testes [Paradidymis/Giraldé’s Organ, Appendix testis and epididymidis, among others (6)], the “Waldeyer White Line” refers to the line connecting the ovary with the peritoneum, the “Waldeyer-Tract” refers to the “dorsolateral fasciculus” on the spinal cord (also known as: Lissauer bundle), “Waldeyer’s fossa” describes a recess which is occasionally found behind the superior mesenteric artery in the region of the mesenteric root (2, 7, 8), the “Waldeyer sheath” which is the tubular space between the bladder wall and the intra-mural part of the ureter. His memoirs appeared in 1920 [Figures 2 and 3; (3)].

## Biography

Waldeyer was born on October 6, 1836 in Hehlen (Kreis Holzminden). He spent his youth in the Paderborner Land region, where his father was an estate manager on Gut Abbenburg (9). He emphasized his descent from a Westfalian farming family with pride (10). The early-recognized talent of the firstborn compelled his parents to allow him to prepare for his university studies at the nearby Gymnasium Theodorianum in Paderborn. The young Waldeyer completed his grammar school time quickly and with ease (10) and in 1856, he obtained his certificate of eligibility for university education. In October 1856, he took up a place in the Universität Göttingen, with the aim of studying mathematics and natural sciences. After two semesters, he came into contact with the anatomist Jakob Henle. He was so fascinated by Henle that he changed to study medicine in 1857. Already at this early time, he had made the decision to become a university professor. Göttingen was the university of the Kingdom of Hanover. However, Waldeyer, who was a Prussian by birth, had to complete his exams in a Prussian university. As a result of this, the political disunity of Germany at that time led to his move to Greifswald where he spent five semesters (10). His interest in the history of evolution, which was not taught in Greifswald at that time, led him to Berlin where he concluded his studies in March 1862 with the State Medical Exam. On July 23, 1861, he obtained his doctorate with the doctoral thesis “De claviculae articulis e functione” which was written in Latin, as was custom at that time (10). In 1862, he moved to Königsberg as Assistant for Physiology and Histology. In 1863/1864, he became engaged to his later wife (née Dillenburger, the marriage took place in 1866). As a Catholic, habilitation at the Universität Königsberg, which was purely Protestant at that time, was denied to him. For this reason, he moved to Breslau as an Assistant at the Physiological Institute under R. Heidenhain in 1864. In 1865, while not quite 29 years of age, he was appointed to the role of non-tenured professor. The non-tenured professorships for pathological anatomy were subsequently converted to full professorships, so he became a full professor in 1867. In 1872, he was subsequently appointed to the full professorship in Strasbourg. The time in Strasbourg was the best time of his life (3, 10). He declined calls to Vienna, Bonn, and Munich (10). In the year 1883, the move to Berlin took place. He remained there for the unusually long period of 33.5 years, as Full Professor for Anatomy and Director of the Anatomical Institute. His great talent as a teacher ensured that his lectures were always filled to the brim (10). In contrast to this, it is curious that his first lecture in Breslau was only attended by three students, in accordance with the old motto “Tres faciunt collegium” (11).

His honorary titles, his honorary memberships of scientific societies and academies, nominations, honors, and accolades are very numerous. During his long tenure in Berlin, he was deacon and rector magnificus several times. In 1912, he was called to the (Prussian) House of Lords. In 1916, on the occasion of his resignation, he received a hereditary peerage: he kept the memory of his mother, née v. Hartz, in his ennoblement (“v. Waldeyer-Hartz”). According to Sobotta, he passed away peacefully after a cerebral apoplexy on January 23, 1921.

“One can only marvel as to how it was possible for a man such as Waldeyer, who was already snowed under with work, to develop such multilateral tasks. He belonged to those lucky characters who do not complain about the amount of work they perform but feel even more fulfilled, the more work they perform” (10).

## Waldeyer’s Fascia

The referencing of the Waldeyer fascia as an anatomical term appears to be incomplete, but from the middle of the previous century, it finds increasing usage in surgical–anatomical and surgical writing, in particular in connection with rectal surgery (2).

Interestingly, the exact morphological substrate is sometimes construed and described differently. According to Waldeyer’s publications, Crapp and Cuthbertson, who have presented the most conclusive and most decided monography to date, assume that he meant and described the entire fascia within the lesser pelvis without actual emphasis of the fascia between the rectum and the sacrum (2, 4). In some anatomical publications, the Waldeyer fascia is understood to be the fascia between the sacrum and the “anorectal junction” (12–14). Goligher understands this to be the thickened parietal endopelvic fascia of the sacrum and the coccyx and its extensions to the anorectal junction (15).

Smout and Jacoby perceive this as the visceral pelvic fascia layer which surrounds the vesico-ureteral junction (2, 16). Last understands this to be the fascia hanging on the lower part of the rectal ampulla which also surrounds the superior hemorrhoidal vessels (2, 17). According to Cunningham’s Textbook of Anatomy, it is the (not usually occurring) fascial layer in the mid-line between the sacral bone and the rectum (18). Wilson describes it as the entire fascia behind the rectum with connections to the caudal part of the broad ligament (of the uterus) and the utero-sacral and infundibulo-pelvic ligaments (2, 19). According to Stelzner, the Waldeyer fascia (parietal internal pelvic fascia) is the fascial layer which lines the entire pelvic funnel and forms the posterior wall of the retro-rectal fiber layer (20).

Today, in relation to Wilhelm Waldeyer, surgical–anatomical publications generally speak of the rectal fascia. However, in the 1st edition of the anatomical textbook “Traité d’Anatomie Humaine” by Poirier and Charpy from 1894, there is an earlier description of the structure by Thomas Jonnesco (or Ionescu) which was further continued in the 2nd edition of the work in an altered form. According to current thought, Jonnesco is therefore the first describer of the structure as “la gaine fibreuse du rectum” (21–23). In his fundamental work, Gerota refers to Jonnesco’s description and equates the terms “gaine fibreuse du rectum” and “fascia propria recti” (24). Jonnesco and Gerota were successive surgical full professors at the University of Bucharest. The Rumanian Textbook Tradition on the subject of rectal surgery, contains therefore, based on Jonnesco’s description, the term of the perirectal fascia as a “teaca rectului Toma Ionescu” (23–25).

Mandache and Chiricuta, the successors of Jonnesco and Gerota, can also be perceived as the “first describers” of the “Circumferential Margin Concept” as they interpreted the perirectal fascia as the macroscopic marginal structure from the direct infiltration of neighboring structures (23, 25).

Should one then ask “Jonnesco versus Waldeyer” (22)? No, because neither had entered into competition with regard to the “first description” of the anatomical structure which is today usually known as “Waldeyer’s fascia.” In contrast: it is fascinating to see how the scientists Gerota, Jonnesco, and Waldeyer were familiar with the work of the others and referred to one another.

Crapp and Cuthbertson were the first to refer to the bifurcation of the anatomical substrate, which is generally perceived to be the Waldeyer’s fascia. According to this, Waldeyer described the upper part of this substrate (“pelvine fascia”), the lower part (“recto-sacral fascia”) was to be interpreted as a separate structure (2). Garcia-Armengol et al. suggest that the recto-sacral fascia arranges the recto-fascial space into an upper and lower part. In their anatomical study, they prove that the “floor” of the *lower* part and the retro-rectal space correspond overall to the term of the Waldeyer fascia. In their opinion, the Waldeyer’s fascia and the recto-sacral fascia are, due to their different topographic connections, completely different anatomical structures (26). Even if the terminologies concerning their different details were not completely clarified in their corresponding definitions, the retro-rectal space was indeed used relatively early by the pioneers of rectal surgery (Miles, etc.) for immobilization of the rectum (20).

From a historical perspective, it is crucial that both Waldeyer and Jonnesco assumed a cylindrical perirectal fascia as visceral sheathing of the rectum as a partition from the sacrum. Further to this, the sacrum is lined with a parietal pelvic layer/fascia. Between these two fasciae, there is an avascular zone which, from a morphological perspective, is the requirement and the main substrate for the TME. This “classical” anatomic-morphological perception was confirmed emphatically, among other things by the works of Havenga et al. as well as of Bissett et al. (23, 27, 28). With reference to the actual and current concept of TME in general, Waldeyer’s role is, on the other hand, problematic as he wrote in his influential publication “The Pelvis,” the mesorectum should be deleted from the anatomical literature (20).

## Chromosomes

Waldeyer’s celebrated publication “Über Karyokinese und ihre Beziehungen zu den Befruchtungsvorgängen,”“About caryokinesis and its relationships with the fertilization processes,” in which the term “chromosomes” was introduced into the terminology and the medical–biological international literature, appeared almost 130 years ago, initially in German and soon afterward in English and French (29, 30). “I would like to permit myself to suggest that the specific technical term *chromosomes* is attributed to those things which Boveri titled *chromatic elements*, on which one of the most important parts of caryogenesis, Flemming’s longitudinal splitting, is performed. They are of such importance that a particularly short name appears desirable. If the term which I suggested can be practicably used, then it will become established; if not, it will vanish into oblivion” (29). In this way, this term still reminds us of a famous anatomist who examined everything which had been written about cell division up to this point and who had worked through the heap of controversial literature on the subject, in order to introduce the term “chromosome” into the nineteenth century world of cytogenetics (31). In this publication, Waldeyer summarized, today one would perhaps say “reviewed,” the experimental and theoretic work of his contemporaries such as Edouard-Gerard Balbiani, Edouard van Beneden, Theodor Boveri, Walther Flemming, Oskar Hertwig, Carl Rabl, August Weismann, Anton Schneider, and numerous others [in total more than 200 references (30)].

The benefit of the discovery of cell division and its main components is due to the Giessener Zoologist Anton Schneider. Until today, many still ask the question how the anatomist and pathologist Waldeyer could become such an authority in the area of chromosome research, especially as he never conducted any experimental research in relation to chromosomes. According to Zacharias, a key to this is the circumstance that Waldeyer was not only a gifted teacher but also an excellent microscopist and microscopic researcher, and therefore was intensely involved in the progress and insights of microscopy—with the microscope as the most modern instrument at the end of the nineteenth century (31). In contrast to most cytology researchers of his time, Waldeyer was convinced that a key theory of “caryogenesis” had not yet been discussed. He emphasized the uncertainties with reference to direct nucleus division (amitosis), the varying viewpoints of the chromosome movement in the mitosis and the problem of inadequate differentiation between mitosis and meiosis. He does discuss the formation of the polar bodies in oogenesis, but he also did not recognize the reduction of the hereditary substance to a simple chromosome set (haploidism) as an absolute requirement for fertilization (31). One must not forget that there was no broad experimentally founded or even logically derived cell theory at that time. Schwann and Schleiden, the founders of cell theory (1838/1839) were strongly convinced that *de novo* cells formed from a structureless substance, the so-called “cytoblast.” Remak und especially Virchow eliminated this perception with their dogma of “omnis cellula e cellula” (1855).

The study of cells, cell biology, and even genetics was only in the early stages at this time. Even now, their birth reads like an exciting crime novel. With reference to the synopsis and evaluation of the theoretic-experimental knowledge and the influence until today of valid terminology at the end of the nineteenth century, Wilhelm Waldeyer is regarded as one of the crucial “obstetricians.”

## Neurons

Waldeyer is classed as the founder of the so-called neuron theory (32, 33). The Greek word “neuron” means “tendon, sinew, ligament; nerve” and is anciently related to and means the same as the Latin word “nervus.” According to today’s perception, Waldeyer’s role in the acceptance of the neuron theory was not straightforward, as there was a list of research sources and also attempts which preferred other terminology (34). The first description is attributed to the Swedish scientist and philosophist Emmanuel Swedenborg: Neuron—a nerve cell with its extensions. Ehrenberg, Remak, Purkinje, Deiters, Schultze, Golgi, His, Forel, Nansen, Cajal, and others delivered significant research results with reference to the nerve cell and the nervous system (34, 35). The scientific methods for this were quite different, as they originated in histological, pathological, functional, and comparative studies. The neuron theory suggested by Waldeyer at the end of the nineteenth century assumed that nerve tissue is built from individual cells, which are genetic, anatomical, functional, and trophic units (35).

The illustrious pioneers of the neuron theory or neuron doctrine were neuro-scientists, clinically active doctors, one polar researcher, and three Nobel prize winners (35). Both the cell theory and also the neuron theory [Waldeyer (32)] are, from a historic perspective, the result of technical and conceptional advancement. Both had to gain acceptance in competition with the dogmata which had been valid until that point. Until the middle of the last century, the neuron based on the cell theory focused on the interneural communication in the field of tension of the Golgi continuity and the concept of contiguity.

In contrast to the cell theory, which is still of the utmost importance in every field of biology, the meaning of the neuron theory fades somewhat when confronted with the current developments of neurosciences (36). At the beginning of the twentieth century, however, the cornerstones of the neuron theory remained stable and credible in view of the new conceptional contributions, for example, from Herrick or Heidenhain (34).

The important and consolidating role of Waldeyer with reference to the neurone theory is not contested from a medical historical perspective, because the influence of his works formalized it (34). “The whole doctrine had been brought to a focus in the celebrated essay of Waldeyer (32)” [Garrison (1914), in Ref. (34)]. As in the case of the chromosomes, this meaning is not only in the contribution of one’s own research results, but also especially in the original and sustainable naming and more still in the conceptional synoptic further development of the current available findings and their consistent propagation. In other words: once again, Waldeyer’s inventive creativity expresses itself in the collection, in further and advanced thinking and in the conceptional further processing of the available scientific results.

## Discussion and Conclusion

Measured on his numerous contributions, some of which are still valid today, Wilhelm Waldeyer can really be considered as a great forefather. Waldeyer also erred and was overtaken by history. His writing on the study of the female is read today as pure anachronism and did not remain without shining contradiction (37). Lina Morgenstern, at that time a famous representative of the German women’s movement, countered: “Highly esteemed Professor! If I attempt to write a contradiction to individual points of your presentation concerning the medical study of women, this will occur from the following aspects: the significance of a presentation lies in the topic which is discussed, in the position which the lecturer occupies in the scientific and civilized world, and in the audience which is being spoken to. In all three directions, your presentation is of the utmost importance for the female movement and should not be underestimated. Especially at this time, as there is a mighty energetic movement in our fatherland, from various female circles, which is demanding female doctors for female and pediatric illnesses as a sanitary and moral necessity and therefore aiming for medical study of women in Germany, the lecture from a famous anatomist (…) seems to be like a declaration of war from the enemy camp: even more so, than the doctors who naturally listened to this speech with great approbation, who are the natural opponents of female study” (38). In his memoirs (3), he somewhat relativizes this view (“I let myself be led…. to lecture on this, in which I generally expressed myself in a hostile manner… I therefore stabbed into a wasp nest… I was more or less attacked…”), respectively, he was educated by the normative strength of the facts (“Later, when women were officially immatriculated and furnished with all academic rights, I had to permit them”). He remained a lifelong opponent of co-education [(3), p. 196 ff.]. From 1908, the way to Prussian Universities was open to German women—a cursory and interesting summary of these labor pains and the associated polemics can be found by Völker (39).

Already during his lifetime, his teaching ability significantly preceded the research capacity. It would, however, be false to overshadow Waldeyer’s merits as a researcher (10). His main scientific merit is in his excellent summarizing interpretations of current questions of anatomy and evolution, which particularly shows his simultaneous gift as a researcher and a teacher.

With great skill, he knew how to process and specify the outlooks and results of individual researchers in the relevant field so that the results were expressed better by him than in the original works. Sharper and more logical specification than the authors themselves was often possible for him, he was particularly happy with this in the choice of new names (10). His last, great, monographic, topographic-anatomical work deals with the pelvis (“Das Becken”). The portrayal of this most important area is counted as one of the most complete which has ever been accomplished in the field of topographic anatomy; it is a real treasure trove of everything scientific, both for the specialist anatomists and for the practical physicians (10).

Waldeyer is an exceptional phenomenon even for the time which was overabundant with illustrious biographers at the beginning of the last century—as a teacher, researcher, specifier, summarizer, and finalizer, and as a medical personality.

## Author Contributions

All authors contributed substantially to this manuscript.

## Conflict of Interest Statement

The authors declare that the research was conducted in the absence of any commercial or financial relationships that could be construed as a potential conflict of interest.
